# Supplementary material for: Content-rich biological network constructed by mining PubMed abstracts
Source: BMC Bioinformatics. 2004 Oct 8;5:147. doi: 10.1186/1471-2105-5-147 (PMC528731; doi:10.1186/1471-2105-5-147)
Supplement: Additional File 2 — The original results of the above study (non-essential files are deleted to keep the file size under the limit set by BMC bioinformatics). [file 1471-2105-5-147-S2.bz2 › chilibotAdditionalFile2/dip20/601ID10052460E10980/html/HOXB1_PBX1.html]

 


 **HOXB1** and **PBX1** 
  
Found 178 abstracts in PubMed, retrieved 20.  
 

 What does Google say? 
 PDF only 
| .edu only 

---

**Interactive relationship** (e.g. stimulation, inhibition, etc)

**Neutral relationship**- In the presence of all three nuclear factors, cooperative interactions between recombinant  **PBX1**  and PREP1 or  **PBX1**  and  **HOXB1**  result in binding of the heterodimers to FPB in vitro.  Ref: 10748126 J Biol Chem, 2000
- DNA binding assays and cell transfection experiments revealed that the bipartite core sequence of FPB 5 ATCAATCA 3 binds the  **PBX1**  2, PREP1, and  **HOXB1**  proteins, and this in turn leads to promoter transactivation.  Ref: 10748126 J Biol Chem, 2000
- This observation implies that this region is at least partially preformed and thus ready to interact with  **Pbx1**  and stabilize binding of  **Pbx1**  and  **HoxB1**  to DNA.  Ref: 11369863 Protein Sci, 2001
- The  **Hoxb1**  autoregulatory element comprises three HOX PBX  [ **PBX1** ]  binding sites.  Ref: 11046157 Mol Cell Biol, 2000
- The  **HoxB1**  hexapeptide is a prefolded domain implications for the  **Pbx1**  Hox interaction.  Ref: 11369863 Protein Sci, 2001
- Like PREP1, PREP2 PBX  [ **PBX1** ]  heterodimers are capable of forming ternary complexes with  **HOXB1** .  Ref: 11972344 Nucleic Acids Res, 2002
- The crystal structure of a human  **HoxB1**   **Pbx1**  DNA ternary complex revealed that interactions between the two proteins are mediated by the  **HoxB1**  hexapeptide, which inserts into a hydrophobic pocket in  **Pbx1** .  Ref: 10448033 J Mol Biol, 1999
- NMR studies of the  **pbx1**  TALE homeodomain protein free in solution and bound to DNA proposal for a mechanism of  **HoxB1**   **Pbx1**  DNA complex assembly.  Ref: 10448033 J Mol Biol, 1999
- Herein we describe NMR structural calculations of the hexapeptide of  **HoxB1**  Nalpha acetyl Thr Phe Asp Trp Met Lys amide that has been shown to mediate binding between  **HoxB1**  and  **Pbx1**  and a hexapeptide consensus sequence Nalpha acetyl Leu Phe Pro Trp Met Arg amide.  Ref: 11369863 Protein Sci, 2001
- The structures of the  **HoxB1**  peptide and the trans form of the consensus peptide reveal a turn very similar to that found as part of the  **HoxB1**   **Pbx1**  DNA complex in the X ray crystal structure.  Ref: 11369863 Protein Sci, 2001
- However, on the  **hoxb1**  autoregulatory enhancer, Hoxa1 and Hoxa1 NA KR may display distinct activity despite the presence of Pbx  [ **PBX1** ] , in a cell type dependent manner.  Ref: 12060683 Nucleic Acids Res, 2002
- The r4 enhancers from  **Hoxb1**  and Hoxb2 have the same activity, but differ with respect to the number and organisation of bipartite Pbx  [ **PBX1** ]  Hox PH sites required, suggesting the intervention of other components sequences.  Ref: 10654609 Development, 2000

**Non-interactive relationship** (e.g. studied together, co-existance, homology, etc.)

- Disruption of the homeobox  [ **PBX1** ]  gene Hoxb  [ **HOXB1** ]  6 in mice results in increased numbers of early erythrocyte progenitors.  Ref: 10996827 Am J Hematol, 2000
- The authors previously have shown that the homeobox  [ **PBX1** ]  gene Hoxb  [ **HOXB1** ]  5 is necessary for normal airway branching during lung development.  Ref: 11101743 J Pediatr Surg, 2000
- We previously showed that  **HOXB1**  and  **PBX1**  activate transcription from the b1 ARE by binding to sequences required for the expression of a reporter gene in rhombomere 4 in vivo.  Ref: 11278854 J Biol Chem, 2001
- In contrast, no change in conformation is observed upon mixing the  **HoxB1**  protein with  **Pbx1**  in the absence of DNA.  Ref: 10448033 J Mol Biol, 1999
- Analyses of the expression and targeted disruption of the homeobox  [ **PBX1** ]  gene  **Hoxb1**  demonstrate that it is essential for patterning progenitor cells along the entire DV axis of rhombomere 4 r4.  Ref: 11076756 Development, 2000
- In man there are 39 homeobox  [ **PBX1** ]  genes of the HOX family in four loci, HOXA, HOXB  [ **HOXB1** ] , HOXC and HOXD on chromosomes 2, and 2.  Ref: 10671062 Hum Mutat, 1998
- We show that, in P19 embryonal carcinoma cells, GCs potentiate RA induced expression of the murine Hoxb 1  [ **HOXB1** ]  gene through an autoregulatory element, b1 ARE, recognized by the  **Pbx1**  and  **HOXB1**  homoeodomain proteins.  Ref: 12487626 Biochem J, 2003
- Analysis of two distinct retinoic acid response elements in the homeobox  [ **PBX1** ]  gene  **Hoxb1**  in transgenic mice.  Ref: 11891985 Dev Dyn, 2002
- Despite the presence of  **HOXB1**  and  **PBX1** , this enhancer fails to activate reporter gene expression in retinoic acid treated P19 cell monolayers.  Ref: 11046157 Mol Cell Biol, 2000
- This study suggests a model for the assembly of a stable  **HoxB1**   **Pbx1**  DNA ternary complex.  Ref: 10448033 J Mol Biol, 1999
- The methods proposed in this paper are then applied to population data at the dopamine D2 receptor locus DRD2 and at the homeobox  [ **PBX1** ]  B HOXB  [ **HOXB1** ]  gene cluster.  Ref: 10738528 Ann Hum Genet, 1999
- Similarly, overexpression of different combinations of  **PBX1** , PREP1, and  **HOXB1**  transactivates FPB driven transcription.  Ref: 10748126 J Biol Chem, 2000
- Therefore, we hypothesize that expression of two additional mesenchymally derived nuclear transcription factors, retinoid X receptor alpha RXR alpha and homeobox  [ **PBX1** ]  b 5 Hoxb  [ **HOXB1** ]  5, would also be altered in hypoplastic lungs.  Ref: 12505043 J Surg Res, 2002
- Association of bronchopulmonary sequestration with expression of the homeobox  [ **PBX1** ]  protein Hoxb  [ **HOXB1** ]  5.  Ref: 11101743 J Pediatr Surg, 2000
- Additional transfection experiments showed that the  **Pbx1**   **HOXB1**  heterodimer was the target for the GC effect.  Ref: 12487626 Biochem J, 2003
- A genetic polymorphism in the human  **HOXB1**  homeobox  [ **PBX1** ]  gene implying a 9bp tandem repeat in the amino terminal coding region.  Ref: 10671062 Hum Mutat, 1998
